# The case for an inclusive scholarly communication infrastructure for social sciences and humanities

**DOI:** 10.12688/f1000research.26545.1

**Published:** 2020-10-22

**Authors:** Maciej Maryl, Marta Błaszczyńska, Agnieszka Szulińska, Paweł Rams

**Affiliations:** 1 Institute of Literary Research of the Polish Academy of Sciences, Nowy Świat 72, 00-330 Warsaw, Poland

**Keywords:** scholarly communication, SSH, open access, open science, research infrastructure

## Abstract

This article presents a vision for a scholarly communication research infrastructure for social sciences and humanities (SSH). The COVID-19 pandemic has highlighted the pressing need to access research outputs without the traditional economic and temporal barriers. This article explores the current scholarly communication landscape, assessing the reasons for the slower uptake of open access in SSH research. The authors discuss such frontiers as commercial interests, sources of academic prestige and discipline-specific genres.

This article defines and discusses the key areas in which a research infrastructure can play a vital role in making open scholarly communication a reality in SSH: (1) providing a federated and easy access to scattered SSH outputs; (2) supporting publication and dissemination of discipline-specific genres (e.g. monographs, critical editions); (3) providing help with evaluation and quality assurance practices in SSH; (4) enabling  scholarly work in national languages, which is significant for local communities; (5) being governed by researchers and for researchers as a crucial factor for productive, useful and accessible services; (6) lastly, considering the needs of other stakeholders involved in scholarly communication, such as publishers, libraries, media, non-profit organisations, and companies.

They conclude that a scholarly-driven, inclusive, dedicated infrastructure for the European Research Area is needed in order to advance open science in SSH and to address the issues tackled by SSH researchers at a structural and systemic level.

## Introduction

“I call on all countries, companies and research institutions to support open data, open science, and open collaboration so that all people can enjoy the benefits of science and research” ( Ghebreyesus, 2020). Although we have heard such statements many times from different actors in many countries, this one was profoundly different thanks to the unique context: it was delivered by the director-general of World Health Organisation, Tedros Adhanom Ghebreyesus, at a media briefing on the COVID-19 pandemic. Furthermore, it was followed by a proposal to create “a pool of rights to tests, medicines, and vaccines, with free access, or licensing on reasonable and affordable terms, for all countries” ( Ghebreyesus, 2020). It put access to knowledge in the right perspective, as a basic human right to access the outputs of scientific research.

The COVID-19 pandemic has deeply influenced our thinking about scholarly communication and the future of open access. It will take time to realise how deep this change is, but one thing is certain: this emergency has forced academics to look differently at the practices they had believed to be indispensable, both in the connections within the scholarly community, and between scholars and the public. The sudden rise in remote meetings and conferences is the best example here. However, this change also affects access to scientific outputs. Big commercial content providers like Elsevier or EBSCO, opened some of their resources to address the issue of students and scholars being stranded at home without library access, as well as to “accelerate the fight against coronavirus,” which proves, indirectly, that paywalls and closed access actually slow down research. This sudden eruption of open access to scientific content proved how indispensable it is for international and interdisciplinary collaboration in addressing the great challenges of humankind. On the other hand, this situation gave additional visibility to open access publishers and repositories, which didn’t have to make their resources public for a limited timeframe as they had already been freely available; and so they concentrated on creating dedicated collections ^1^ and guidelines ^2^ about the open content. 

It is against this background that we sketch the scientific case for the future infrastructure for open scholarly communication. Every discussion of the future should be deeply rooted in the past in order to avoid the fallacy of the status quo, i.e., treating the current situation as a given, and every novelty as unprecedented. This is very true for the scholarly communication that evolved throughout the centuries together with technological means. We will thus follow the recent EC report *Future of Scholarly Publishing and Scholarly Communication*, which proposes to define scholarly communication broadly as “any form of exchange used by scholars and researchers to participate in the elaboration of knowledge through critical discussions and conversations with fellow humans. This encompasses all the procedures, from the purely informal conversation to the highly formalised stage of ‘publishing’” ( Expert Group to the European Commission, 2019, 14). In this approach scholarly publishing is a *formalised* sub-set of scholarly communication. This perspective allows us to look beyond technology to the source of current communication practices: platonic dialogues, debates, treaties, and letters. Similarly, we need to perceive modern research infrastructures as new versions of the traditional ones, like libraries, archives, and museums, which have been serving the main purpose of facilitating research and knowledge exchange by using available technological means.

The digital transformation brought about new opportunities for creating, sharing, discovering, and accessing scholarly resources. Although we have seen a gradual shift towards open practices, some disciplines—notably social sciences and humanities—seem to lag behind ( Ferwerda *et al.*, 2017, 19). It appears that further progress, as well as acceleration, requires a dedicated, inclusive infrastructure that can streamline the fragmented initiatives, address the specific challenges faced by these disciplines, and attune the services to their particularities. In other words, we need to “critically appraise what we need from a scholarly communications infrastructure and to simultaneously build pragmatic and non-damaging transition strategies to harness the full power of open, digital dissemination” ( Eve, 2015, 15). This is a pertinent issue in the light of the European Commission’s strategy to create a European Open Science Cloud, a service enabling European researchers to “store, share, and re-use data across nations and scientific disciplines through the open science cloud and without leaving their desk” ( Burgelman *et al.*, 2019). This calls for a coordinated effort on the part of social sciences and humanities (SSH) researchers, and for infrastructures to achieve a proper representation of disciplinary needs in this new landscape. A research (and researcher-driven) infrastructure for scholarly communication in SSH should respond to these challenges and opportunities, and enable scholarly communication that would take full advantage of digital affordances, but would not forget its core values.

## 1. Scholarly communication: from theory to infrastructure

### 1.1. Communication as the enabler of science

***Definition of scholarly communication*.** The term *scholarly communication*, in the narrow sense, is usually employed to describe traditional and institutionalised publishing practices as “the system through which research and other scholarly writings are created, evaluated for quality, disseminated to the scholarly community, and preserved for future use” ( ACRL Scholarly Communications Committee, 2006). Yet, as the definition in the introduction has highlighted, formalised publishing practices are just a subset of a larger pool of various communication practices, which would entail, for instance, all sorts of formal and informal communication through various channels like emails, social media, blogs, press, etc., both between scholars and between scholars and the public. Thus, apart from researchers, the group of potential actors involved in scholarly communication includes “students, educators, policy makers, public administrators, funders, librarians, journalists, practitioners, publishers, public and private organisations, and interested citizens” ( Kraker *et al.*, 2016, 2).

Moreover, communication, conceived broadly, “includes both the dissemination and access to scholarship and research in a variety of formats and states of completion, such as published books or journal articles, research results and data sets, and drafts of papers” ( Husain & Nazim, 2013, 405). While it is true that the relationship between communicating and publishing becomes “increasingly muddy” ( Edmond, 2020, 4), traditional publishing practices still remain the established sources of academic prestige and suppress the engagement with innovative solutions. We aim to show how artificial these distinctions are by discussing the role of scholarly communication in actual research practices.

***The research lifecycle*.** In an often-referenced paper that contributed to the modern understanding of humanities research, John Unsworth (2000) proposed a tentative set of scholarly primitives, i.e., “self-understood” terms describing scholarly activities: discovering, annotating, comparing, referring, sampling, illustrating, and representing. Although this list was not meant to be exhaustive, one omission is striking, namely, the exclusion of communicating. It is even more visible once one realises that all the examples Unsworth provides in the paper for comparison (Babble), linking (Blake Archive), and sampling (VRML visualisation of Dante’s Inferno), have an indispensable communication component attached to them. Perhaps communication is even more *self-understood*, and thus becomes transparent and eventually omitted. To use Unsworth’s nomenclature, communication takes advantage of the additive characteristics of scholarly primitives and enters into combinations with all other scholarly primitives, i.e., it proves to be essential across all stages of research workflow. This means that dissemination should no longer be perceived as the final stage of a research process, running somewhat separately from the actual research, but should be treated as an integral part of all scholarly activities (cf. Giglia, 2019; Nielsen, 2013).

The process of scholarly activities is usually presented as a lifecycle, comprising such activities as discovering/collecting, organising, annotating, analysing, and disseminating (see: Dallas *et al.*, 2017, 4). If we take a closer look, we see how deeply communication practices are embedded in those processes: literature review, data discovery, communication to peers, peer review, editing, dissemination, marketing, and quality assurance (see: Mounier, 2018, 301). Communication is thus an integral part of all stages of a researcher’s workflow, and each of these stages has “specific communication needs that can be addressed by proper services” ( Mounier, 2018, 301). Different stages of the workflow are linked to different operations and software, as evidenced in the *Innovations in Scholarly Communication Survey* conducted by Utrecht University Library. As Edmond and Romary point out, “[a]t certain stages of the research process, it is often not as important to produce an in-depth scholarly summation so much as to provide short snapshots of an experiment’s current developments (as in the hard sciences), or an analysis of a source (in the humanities)” ( Edmond & Romary, 2020, 65). Each phase may entail the use of different formats to communicate early results, observations, data, annotations, etc.

Researchers assume different roles at different steps of this process. They are not only authors, but also information-seekers, annotators, bibliographers, editors, reviewers, etc. These roles are very important for research but do not carry the same level of prestige in academia as the role of author. This has a negative impact on introducing and sustaining changes in scholarly communication ( Edmond, 2020, 13). However, such roles, and the scope of responsibilities attached to them, are constantly evolving because of the imprint that emergent technologies and their applications leave on scholarly communication. For instance, Jason Priem predicts, “the reviewer will metamorphose from gatekeeper to interlocutor and collaborator” (2013, 438). This is also true for institutional actors in scholarly communication, who start to enact new roles, such as repositories that already serve some of the functions traditionally reserved for publishers.

***Communication and the community*.** Communication is thus not only an important factor, but the actual enabler of scholarship. The development of the postal network during the sixteenth century allowed for unprecedented knowledge exchange between scholars within (and often beyond) Europe, forging the idealistic notion of the republic of letters: a common, intellectual space ( Hotson & Wallnig, 2019, 7–11). Within the scientific dimension, these letters allowed for the dissemination of important intellectual breakthroughs, becoming prototypes for the first learned journals in the seventeenth century (ibid., 8). This example illustrates Nielsen’s point that the science remains tentative until communicated: “the meaning of scientific knowledge is not only established by its internal qualities or the method by which it has been produced, it also depends on what other scientists make of it, that is, how scientific knowledge is being communicated” (2013, 2071). Consequently, communication is what constitutes science.

Communication presupposes the existence of a community, where knowledge is exchanged and negotiated leading to a “common narrative and meaning” ( Neylon, 2017a, 3; see also: Nielsen, 2013, 2068). Such communities, whether we call them “thought collectives” ( Fleck, 1979), “epistemic cultures” ( Knorr-Cetina, 1999), or “interpretive communities” ( Fish, 1980), share certain beliefs, concepts, and practices for evaluating new knowledge. We can observe these at different levels of organisation, from small research teams through to scholarly associations, disciplines, or groups of disciplines (e.g. SSH). However, such communities have one thing in common: scholarly communication is essential for fostering their group identity ( Hartley *et al.*, 2018, 6).

Nielsen likens communication to “knowledge travel,” a movement of knowledge between communities, as “[s]cience fundamentally is a shared form of knowledge, and conventional ways of communicating among peers and across epistemic boundaries are central to the collective and collaborative character of science” ( Nielsen, 2013, 2070). Hence, we are able to observe scholarly communication both within a community and between communities, which Kulczycki defines as “external communication science” (the “process of explaining and popularising academic research” to non-scientists), and “internal communication science” (“communication of scientists with scientists”) (2013, 5–6). Fleck suggested a spherical understanding of scientific communities, with “specialised experts” at the core, surrounded by “general experts,” who, in turn, border onto “educated amateurs,” with all encircled by the general public ( Fleck, 1979, 111–12). Elsewhere he notes that, paradoxically, the more widespread the knowledge is the more exclusive it becomes due to its complexity and specialisation. The accessibility of knowledge calls for its constant translation by scholar after scholar, adapting it and interpreting within their own research and thus making it more understandable for the general public or specialists from other areas ( Fleck, 1979, 109). This process of “knowledge travel” and “translation” between different epistemic cultures is especially important for inter- and transdisciplinary endeavours, serving as a bridge that allows for the building of shared understandings and joint research agendas.

So, effective communication would ensure a constant, bi-directional flow of ideas and expertise between those circles and communities. The lack of such communication impedes the innovative and inclusive potential of those collectives ( Kraker *et al.*, 2016; Okune *et al.*, 2018). This could lead to the creation of disciplinary siloes, “knowledge clubs” that operate on the basis of the “joint production and consumption of scholarly output by the scholarly community,” whereas “knowledge is most intensively produced at group-boundaries, in interaction with other, competing groups” ( Hartley *et al.*, 2018, 5; cf. Neylon, 2017a, 5–6). Scholarly communication without the actual community of peers exchanging ideas is reduced to the outward signs of academic prestige, but devoid of its communitarian substance that only a vibrant space for exchange of knowledge can create. This is where “predatory” or “deceptive” publishing flourishes, encompassing a range of problems of academic publishing, from poor content quality to deceptive journals ( Eriksson & Helgesson, 2018).

### 1.2. Open scholarly communication in Europe

“An old tradition and a new technology have converged to make possible an unprecedented public good;” thus begins the Budapest Open Access Initiative (BOAI), signed in February 2002, and hinting at the recreation of the *old tradition* of the republic of letters in the digital environment. Now, almost two decades later, the situation has somewhat improved, but the appetite of open access proponents has also increased, crystallizing in a movement for open science.

Recent initiatives by various institutions reflect the urge to speed-up this transition. The European Commission’s working document that lays the foundations for the next framework programme, Horizon Europe, has identified “rather limited progress at the EU level in the transition towards open science, including on open access to research output” ( European Commission, 2018, 104). The EC adopted a “holistic policy to Open Science,” engaging stakeholders in key aspects ranging from open publications to research data and interoperable services ( Burgelman *et al.*, 2019). For instance, Horizon Europe is to “fully embrace and support Open Science as the new research modus operandi,” including support for open data, FAIR principles, financial incentives, and lack of funding for hybrid journals (European Commission, 2018, 105–6). On the same note, Plan S, proposed by a coalition of funders, explicitly states that starting from 2021 all outputs of grants provided them must be available in open access without embargo. On a global scale, UNESCO has launched The Global Consultation on Open Science, to ensure “a better distribution and production of science in the world”. These actions are aimed at stimulating the communities in its transition to open access.

BOAI had famously advocated “open-access to peer-reviewed journal literature,” either through self-archiving or “a new generation of open-access journals,” which are now commonly known as green, gold, and diamond open access (BOAI). Open science, with regard to scholarly communication, currently has a much wider scope as it applies to all stages of the research process, “from designing the question and methods, to collecting and analysing data, through to the communication and dissemination of findings” ( Hillyer *et al.*, 2017, 18; cf. Krlev, 2019). So, in other words, all elements of scholarly communication discussed above should be freely accessible. Simply (and broadly) put, open access “refers to the removal of price and permission barriers to scholarly research” ( Eve, 2014, 3), with the underlying assumption that everyone should have unrestricted access to knowledge ( Tennant *et al.*, 2016, 15).

There are many arguments in support of open science, which Ulrich Herb (2010) helpfully categorised into groups: science–related arguments (improving scientific communication), financial arguments (response to rising subscription prices), social arguments (reducing the digital divide, fostering societal impact), democracy–related arguments (enabling participation), and socio–political (or moral) arguments (levelling disparities). But still, despite these arguments, recent reports note there are obstacles and slow progress affecting the open access (OA) movement. A recent review of the academic, economic, and societal impacts of OA has concluded that although “Open Access supersedes all potential alternative modes of access to the scholarly literature through enabling unrestricted re-use, and long-term stability independent of financial constraints,” it is still endangered by competition in the unregulated scholarly publishing market ( Tennant *et al.*, 2016, 1–2). The uptake of OA policies in Europe varies from country to country, which will be discussed briefly using a handful of examples ^3^.

In Poland, the 2018 report on the implementation of OA policies reveals that the fragmentation of open science efforts—such as the rise of many small repositories that are not linked to each other ( Szafrański, 2019, 11)—calls for a stronger central coordination ( MNiSW, 2018). The number of institutional OA mandates is small but growing, and reports suggest that the infrastructure is fragmented and in need for more coordination ( MNiSW, 2018; Sójkowska & Gruenpeter, 2019; Święćkowska, 2013). The studies show that the most successful repositories belong only to those institutions where self-archiving is compulsory, but these are still in the minority ( Święćkowska, 2013, 8). Similarly, the Ministry of Science and Higher Education in Poland has focused on giving general recommendations rather than on setting up compulsory, nationwide requirements ( Gowin, 2017; MNiSW, 2015).

In the same way, there is still not enough awareness of the benefits associated with the transition to open access in Greece, and the engagement of key stakeholders has been relatively low ( Picarra *et al.*, 2015). Even in cases where open access platforms (such as Greece’s EKT ePublishing platform, discussed later in this paper) have been adopted, the full transition to online editorial processes has not necessarily followed ( Sachini *et al.*, 2009). Despite the OA initiatives, the majority of scholarly communication efforts in Greece are still market-driven.

Conversely, France remains one of the leaders in terms of national coordination and support for open science initiatives (such as HAL repository), which perhaps stems from its centralised tradition, with the National Centre for Scientific Research (CNRS) as the focal point for scholarly activities. OpenEdition is its signature national infrastructure for SSH (see: MESRI, 2018). In 2019, the National Open Science Fund was created to support research infrastructures, digital platforms, and initiatives concerning open journals and books. One may still note the tension between the private and the public publishing sectors however, with the former opposing OA and the latter embracing it ( Ferwerda *et al.*, 2017, 74–78).

In Croatia, the Ministry of Science and Education is currently adopting the open science agenda and although there are other strategic documents addressing OA, funders and policy-makers still haven’t developed key top-down policies. Numerous OA and OS initiatives have emerged in Croatia and have sought to alleviate this lack of national policies, and to provide the infrastructure necessary to support openness, such as the Croatian Scientific Bibliography (CROSBI), and HRČAK, a common platform for OA journals. Over half of the Croatian OA journals are from SSH disciplines, mostly following the Diamond OA route without article processing charges, grounding their business models on government subsidies. Research and higher education institutions can establish their own institutional and disciplinary, or thematic repositories free of charge via the national infrastructure for digital repositories, DABAR.

In recent years open science has been strongly supported in Switzerland. Since October 2017, the Swiss National Science Foundation (SNSF) has required researchers to include a data management plan (DMP) in their funding application to most of the funding schemes. As of 2020, all results have to be made available in open access. In April 2020, the State Secretariat for Education, Research and Innovation (SERI) agreed with various stakeholders to introduce a national strategy for open research data in 2021 ^4^. In this landscape the social sciences in Switzerland have been supported by the Swiss Centre of Expertise in the Social Sciences (FORS) infrastructure, whereas humanities are under the lead of the Data Service Center for the Humanities (DaSCH); both institutions are supervised by the Swiss National Science Foundation (SNSF).

Although the whole open access landscape has not yet been thoroughly researched and analysed in Luxembourg, it is worth mentioning some significant top-down initiatives, such as the Policy on Open Access adapted by FNR (the National Research Fund), as well as its Open Access Fund programme (focused on promoting OA in projects receiving FNR funding). Moreover, the Scientific Monographs programme for printing books (RESCOM) covers the OA-related costs.

These examples suggest that, despite the long history of the movement, the uptake of open science is still in need of a major boost. Moreover, progress in various European countries is uneven, which calls for more coordination at the EU level. However, there is a key obstacle impeding this transformation, which Martin Paul Eve calls “digital economics,” i.e., “the economics of scholarly publishing in the two interlinked senses of an ‘economy’ of academic prestige and of finance” ( Eve, 2014, 16, 43–85). As for the financial side, academic publishing became a profitable business, functioning in an oligopoly of big commercial companies offering access to scholarly content at very high prices. This concerns the cost of both individual access and library subscription (see: Eve *et al.*, 2017, 122). A good example indicating that something is not right is that access to a single scholarly article tends to cost more than a monthly subscription to a streaming service with blockbusters and popular TV shows. For instance, in order to access some recent work on scholarly communication (e.g. Chisita & Chiparausha, 2019; Sotudeh *et al.*, 2019; Wang *et al.*, 2019; Young & Brandes, 2020) without an institutional subscription, authors would have to pay USD 41.95 per PDF (at the time of writing this article). This is more than the price of many printed monographs, which poses an actual barrier, not only for independent scholars and the non-academic public, but also for institutions that cannot afford costly subscriptions.

The recent report *Untangling Academic Publishing* ( Fyfe *et al.*, 2017) reconstructs the history of scholarly communication, which has become increasingly commercialised since World War 2 in response to the professionalisation of academia and internationalisation of research: “The older model of academic publishing practised by learned societies and university presses had prioritised the wide circulation of high-quality scholarship, with little or no expectation of making money. The new commercial model demonstrated that, in the new world order, it was possible not merely to break even but to make profit” ( Fyfe *et al.*, 2017, 10). This commercial strategy entailed setting up new journals, selling their content to institutions (as they can be charged more) and focusing on the international market to address a larger audience ( Fyfe *et al.*, 2017, 10). Digital distribution only strengthens this model, reducing the actual service cost and allowing archival issues to be charged for on the basis of constantly renewed subscriptions, rather than one-time payments for physical copies. Moreover, it also allows the most popular journals to be bundled together with lesser-known ones in order to increase the breadth (and price) of institutional subscriptions. Thus, the profits of large commercial scholarly publishers are based on the unpaid labour of authors and reviewers, as well as the revenue from public funding either in the form of subscriptions or article-processing charges.

This model has been criticised due to the immense subscription costs that make access to resources uneven, limiting the transfer of knowledge to the community ( Kraker *et al.*, 2016). This is also a concern in developing countries, which struggle to foot the bill for access prices ( Arunachalam, 2017; Tennant *et al.*, 2016). Another issue is that the money spent on access to paywalled content could be used instead for the benefit of scholars: “it means less support is going back into our library communities and that we have less agency in the future development of scholarly communication infrastructure” ( Wipperman *et al.*, 2018, 244).

These economic barriers are even more puzzling if we consider the cost structure of digital dissemination. Eve points out that OA entails “nonrivalrous commodity exchange,” in which “the ‘use’ of a commodity does not entail somebody else’s inability to use it,” so there is no cost attached to reproducing the object ( Eve, 2014, 16). In other words, there is no particular cost associated with a single article download. And acquiring a digital copy of an article surely does not impede somebody else’s ability to do the same, which makes the high prices even less justified. There are many successful OA business models for scholarly publishers that allow for a scholarly-led spending of public resources (for an overview see: Milloy *et al.*, 2012; Speicher *et al.*, 2018; Withey *et al.*, 2011), but the dominant position of the big commercial players keeps them fragile and difficult to sustain (e.g. Björk, 2017), and slows down wider change. Moreover, even if the OA practices are adopted by big commercial providers, there is still a risk “that a small number of companies will own most of the critical data assets, analytics, and platforms used by the scientific community” ( Aspesi & Brand, 2020, 576), thus maintaining the dominant position.

A second barrier, closely linked with the financial aspects, is that of academic prestige. Ulrich Herb, who analysed the uptake of OA through the lens of Bourdieu’s concept of scientific capital, concludes that although “the moral vibrancy [of OA] is overwhelming,” scholars are concentrated on accumulating such capital, which is usually achieved through publishing in journals with a high impact factor (2010). Similar conclusions could be drawn from the analysis, by Fyfe *et al.* (2017), of the history of the relationships between commercial actors, academic prestige, and scientific communications. They conclude that prestige in academia is currently linked with “traditional forms of academic publishing, many of which are controlled by commercially-motivated firms” ( Fyfe *et al.*, 2017, 18). The sad outcome is that there are no “credible, prestige-generating alternatives” and that the players in the online publishing business tend to see “online publishing as a valuable income stream, rather than seeking ways to use the potential of the Internet to carry out their traditional ideals of promoting the circulation of knowledge” ( Fyfe *et al.*, 2017, 18).

It should be noted that economic observations and trajectories stem from the analyses of the ‘global West’. A slightly different case could be made for national communities where the scholarly communication is conducted in local languages and with a strong tradition of state-funded publishing, such as Poland and other countries of the former Eastern bloc. Apart from the issues discussed earlier, like high subscription rates, we also observe a diminishing position of the local journals. For instance, the Polish evaluation system for journals is based on the Scopus database which has an uneven, to say the least, representation of scholarship in local languages and discipline-specific outputs, including monographs. If local journals and monographs are not indexed, the impact of a publication could be measured solely on the basis of the international reception, what gives a false impression of a lower impact especially in the case of humanities (e.g. Polish studies). Thus, the prestige of a publication became closely linked with a business product with opaque inclusion policies beyond the control of the scholarly community. This is a dangerous situation as it creates a clear conflict of interests for a company which both publishes journals and ranks their impact. Hence, the pressure for internationalisation, aptly described by Kulczycki & colleagues, (2019b), has a clear business dimension with global corporations benefiting from state policies, which are at the same time suppressing the local high-quality journals.

So, although there seems to be a consensus that OA is generally good for research, the broader adoption of open practices will not happen without a substantial transformation of the practices of prestige allocation in academia. Eve describes the systemic aspects of this mechanism: since the venue’s prestige is used as a proxy measure for quality, it encourages publication in acclaimed outlets and has a cooling effect on innovation, while conservative evaluation mechanisms petrify this system ( Eve, 2014, 50). And, according to Edmond, it is a “generational fallacy” to believe that the new generation of scholars will bring change to academia, as the proxies for prestige are also used for funding allocations and job searches, so the system reinforces itself ( Edmond, 2020, 8). Instead, we need systemic action that can address the system of scholarly communication economics in its entirety, addressing both business models, and prestige.

To sum up, the European Commission tries to address these challenges through its ‘holistic approach’, a top-down measure, which needs to be met by a bottom-up movement towards open science. Robert-Jan Smits, the former European Commission’s Special Envoy for Open Access observed that “[a]lthough researchers all say that they are supporting open access, their dream is still to publish in the most prestigious journals with the highest impact factor, which are often subscription journals. And the universities are obsessed by the traditional rankings using mainly one metric – number of publications in high impact journals” ( Smits, 2018). That is why a comprehensive approach towards open science should address the issues of perceived academic prestige, which can differ across communities of practice and thus require different remedies. A proper, sustainable, and far-reaching response to these challenges requires a synergy of initiatives conducted by various actors at different levels, which could be provided by a research infrastructure. The recent actions undertaken by the European Commission, which have led towards the creation of the European Open Science Cloud (EOSC), are meant to put those ideas into practice. The EOSC Declaration, widely adopted by numerous European institutions, research infrastructures (RIs), and societies ^5^, sketches out a vision of a pan-European meta-infrastructure “federating existing resources across national data centres, European e-infrastructures and research infrastructures” ( European Commission, 2017, 3). The key to the EOSC is its unique governance model, which intertwines community-driven and multi-governmental movements ( Budroni *et al.*, 2019, 130–31). Thus, it is essential that the needs of scholarly communication are properly addressed by the relevant communities, allowing for the proliferation of open science. The EOSC seems to be the ultimate embodiment of the ‘holistic approach’, as it recognises that OS can be achieved only through “considerable cultural change,” in which “no disciplines, institutions, or countries must be left behind” ( European Commission, 2017, 1).

### 1.3. Inclusive, discipline-specific knowledge infrastructures for scholarly communication

The European Commission defines research infrastructures broadly as
facilities, resources and related services that are used by the scientific community to conduct top-level research in their respective fields and covers major scientific equipment or sets of instruments; knowledge-based resources such as collections, archives or structures for scientific information; enabling Information and Communications Technology-based infrastructures such as Grid, computing, software and communication, or any other entity of a unique nature essential to achieve excellence in research. ( European Commission, 2010, 10)

The authors of the European Science Foundation (ESF) Report on research infrastructures in digital humanities insist that “an RI cannot be defined in an abstract, absolute and immutable way; rather, it is a term that is adapted for and by different disciplines” ( Moulin *et al.*, 2011, 5). The humanities, for instance, have a long tradition of physical infrastructures: “Archives, museums, galleries and libraries have always housed collections of physical objects such as archaeological fragments; paintings or sculptures; inscriptions or manuscripts; books and journals” ( Moulin *et al.*, 2011, 4). All of them have been through the digital transformation and adapted to modern scholarly needs, and adjusted to particular research communities. Thus, the crucial difference between e-infrastructure and research infrastructure is the latter’s situatedness in a particular research community, responding to disciplinary research needs (see: Duşa *et al.*, 2014, 21–39), whereas the former addresses more generic needs; as in the case of OpenAIRE, which provides services for institutional repositories of all disciplines ( Artini *et al.*, 2015).

In order to perform its role for the community, a research infrastructure should be inclusive, i.e., facilitate the work of diverse kinds of actors. Okune *et al.* propose the term “inclusive knowledge infrastructures” to define “tools, platforms, networks and other socio-technical mechanisms that deliberately allow for multiple forms of participation amongst a diverse set of actors” ( Okune *et al.*, 2019, 2). This inclusivity is crucial for reconfiguring power relations and involves diverse communities in knowledge-creation processes ( Okune *et al.*, 2019, 7). In the case of scholarly communication, that would mean a broader pool of actors and all types of scholarly outputs, beyond the refereed ones ( Ren, 2013, 745).

In the following section we will look at the existing scholarship and will try to reconstruct the actual needs of SSH scholars with regard to a scholarly communication infrastructure.

## 2. What SSH researchers need

### 2.1. Specificity of scholarly communication in SSH

Paradoxically, the vocal proponents of open science emerged from the humanities; but the uptake of OA in SSH is still lower than in science, technology, engineering, and mathematics (STEM) disciplines ( Ferwerda *et al.* 2017, 19). Moreover, different models of funding between STEM and SSH influence their opportunities to engage in particular open access practices, with SSH researchers usually having less chances to receive funding for article processing charges (APCs) ( Rowley *et al.*, 2017, 1204). This discrepancy in the humanities, Eve observes, is often explained as stemming either from different communication practices of those disciplines, or the lack of a critique of their own disciplinary communication practices ( Eve, 2014, 24). Suber (2005) listed several differences, arguing that, apart from funding difficulties and less need for immediate access to results, SSH differs in terms of its major communication format, which is the monograph.

Monographs occupy a special place in humanities scholarship. They manifest a crucial specificity of the humanities communication paradigm as a more discursive format, serving different functions to journal communication, allowing for a thick description and complex narrative ( Crossick, 2015, 13–14). As Geoffrey Crossick contends in his study on open access monographs, “[t]he process of constructing and writing a book is often a core way to shape the ideas, structure the argument, and work out the relationship between these and the evidence that has emerged from the research process” ( Crossick, 2015, 3). Moreover, and paradoxically, this complexity may contribute to SSH scholars’ reluctance to share publications in OA, which Fitzpatrick connects with a fear of the wider, non-specialised audience: “[t]he world at times fails to understand what we do and, because our subject matter seems as though it ought to be universally comprehensible ( *You’re just writing about books, or movies, or art, after all!*), readers often are not inclined to wrestle with the difficulties that our work presents” ( Fitzpatrick, 2012, 353). Another crucial issue is that, unlike STEM, SSH tends to be deeply rooted in local contexts and languages. As Mounier puts it, “the diversity of publication venues reflects the epistemic diversity of SSH communities that need their own ways of communicating knowledge to diverse audiences” (2018, 302).

However, it would be a mistake to treat SSH and STEM as completely different, as separated academic worlds that compete with each other for the legitimacy of performing the true and “right” kind of research. As Edmond, Bagalkot, and O’Connor suggest, instead of seeing polarities, we need to perceive these disciplines on “a sliding scale of ‘epistemic cultures,’ which, like human cultures, blend with and branch from each other in a wide variety of modes at a number of border regions” (2016, 2–3). The ongoing project on interdisciplinary practices in Europe (SHAPE-ID) tries to answer the question of why SSH is poorly integrated in inter- and transdisciplinary (ID/TD) endeavours. Early results have identified the root of this problem “in a lack of understanding by researchers, policy makers and funders, about what the [arts, humanities and social sciences] are and what these disciplines can contribute to solving societal problems” ( Vienni Baptista *et al.*, 2020, 14). The authors also observed that these disciplines are invisible in certain contexts and fields ( Vienni Baptista *et al.*, 2020, 19), and have analysed the systemic and institutional obstacles that hinder such cooperation. Bibliometric analyses performed in this study have clearly indicated a certain self-referentiality in the discourse on ID/TD efforts within arts and humanities, and to a lesser extent, in social sciences ( Vienni Baptista *et al.*, 2020, 63), leading to disciplinary siloses that hinder cooperation.

Recognising the potential of SSH would contribute greatly to the European Union’s Grand-Challenges’ approach, which entails defining strategic missions that should be addressed by long term, transdisciplinary research programmes ( Mazzucato, 2018). A timely example of such a contribution, though on a smaller scale, is the OPERAS’ response to the COVID-19 crisis. While STEM disciplines collate data and papers relevant to fighting the disease and finding a cure, the Beyond COVID-19 project aims to create an annotated bibliography of SSH outputs relevant to the pandemic so the actors involved in policy measures have easy access to scholarly outputs that could help them understand the societal and cultural impacts of their decisions. A dedicated infrastructure for SSH could have a positive effect on bridging various disciplines through scholarly communication, and maintaining the visibility and accessibility of SSH outputs that should become an integral part of such missions to achieve better impact. We will now look at the actual needs of SSH scholars with regard to scholarly communication in more detail.

### 2.2. Digital transformations

There is something distinctive about the research process in the humanities. On the basis of interviews with scholars, Edmond *et al.* noted the “nomadic nature of the humanistic knowledge creation process, with its constant refreshing of sources and inspirations” (2016, 6). They pointed out the uniqueness and individuality of scholarly apparatuses ( Edmond *et al.*, 2016, 10–11), which are currently met by digital tools and methods, that remediate all stages of scholarly workflow, from discovery to publication.

This transformation is particularly visible in the development of digital humanities (DH), i.e., an approach being taken up across all disciplines of the humanities and social sciences, which incorporates digital tools in research workflows ^6^. DH focuses on both the production and analysis of digital-born or digitised data, as well as on the implementation of digital information and communication technologies during the different stages of the research process, as evidenced in a Taxonomy of Digital Research Activities (TaDiRAH) ^7^ and the methods ontology proposed by the Network for Digital Methods in the Arts and Humanities (NeDiMAH) ^8^. These remediated operations include the capturing of sources (discovery), creation (e.g. writing, programming), data enrichment by annotating or editing, storage (archiving), and various forms of dissemination, that is, from collaboration, to commenting (reviewing), crowdsourcing, publishing, and sharing.

The uptake of these methods is versatile as evidenced in numerous quantitative and qualitative studies that have analysed the nature of digital methods and tools in the humanities and social sciences (e.g. Antonijevic, 2015; Edmond *et al.*, 2016; Hughes *et al.*, 2015). The *digital turn* stimulated the rapid changes in these areas across all disciplines in a twofold manner: as cutting-edge digital-humanities work, pushing the boundaries of the state-of-the-art; and digitally-enabled research, characterised by the selective use of particular tools by a largely non-digital community to answer a specific, disciplinary research problem ^9^. For instance, the massive use of electronic communication and social media by research communities facilitated collaborative annotation, writing, and the evaluation of scientific argumentation by a community that connects authors, readers, and reviewers, thus creating a productive environment for the emergent publishing initiatives ( Ren, 2013, 745). Scholarly blogs became *creative catalysts*, offering an opportunity to reach multiple audiences and to receive quick feedback on early findings ( Kjellberg, 2010). Such *open publishing* initiatives that allow for open commentary early in the publication process have an immediate benefit for the quality of research through tapping into a larger number of reviewers and the broadest scope of expertise. This “open system,” as Xiang Ren calls it, does not discredit the principle of peer review but instead makes an attempt “to reorganize and democratize” it through “expanding the scale of ‘peers’ and making it more transparent” ( Ren, 2013, 747). In this sense, open access can be seen as a chance “to systematize direct scientific communication,” which does not need formal venues, and instead takes place within specific infrastructures such as archival depots, platforms, personal websites, etc.” ( Mayeur, 2017, 75–76).

Novel means of writing, co-authoring, team-collaboration, and versioning of scientific content have sparked the creation of new genres of scientific communication that allow for a better connection between the thought and its expression. These open and innovative communication practices give an insight into researchers’ thought processes, methodology, and spontaneous discussions instead of just the formal results of their work ( Ren, 2013, 745). The use of the web for disseminating one’s research results also allows authors to experiment with different forms of scholarly communication (often a tweet, an infographic, a blog post, or an extended comment might be more appropriate for conveying certain messages): “What the journal did for a single, formal product (the article), the Web is doing for the entire breadth of scholarly output” ( Priem, 2013, 437).

Open access to research publications, strengthened by robust discovery tools, enables access to vast resources across disciplines, fields, and national/linguistic boundaries, stimulating knowledge exchange and advancement, all of which has clear benefits for the scholarly community. The discoverability of open access resources is crucial, since one of the main goals within the field of scientific communication is to “enable research to be carried out effectively and efficiently” ( King, 2019, 2). Furthermore, the proponents and signatories of the recent *Helsinki Initiative on Multilingualism in Scholarly Communication* highlight the role of access to multilingual resources, which allows for more voices to be heard; it also ensures a wider scholarly and societal impact, which will be discussed in greater detail later in this paper. So, what are the actual needs for a scholarly communication infrastructure in the humanities?

### 2.3 Meta-research on SSH scholarly practices

User-centred research, providing information about users’ actual needs, is key in designing new products or services, as it “can reduce the potential for poorly designed or misused products” ( Lofthouse & Lilley, 2006, 741). Otherwise, there is no guarantee that the infrastructure will address the existing needs of a scholarly community, or that scholars are going to use it (see: van Zundert, 2012; Warwick *et al.*, 2008). The user-centric approach should also be applied to designing a future scholarly communication infrastructure, and researchers as its users with unique habits, needs and expectations ( Kemman *et al.*, 2014, 3). Such needs pertain to all stages of the research lifecycle and users expect infrastructures to support the whole research process ( Boukhelifa *et al.*, 2018; Dallas *et al.*, 2017).

These issues are captured by research on digital practices in the humanities (meta-research), which does not focus solely on the advanced proponents of digital methods, but also analyses digital needs and competences of the SSH community, allowing for the transfer of skills and knowledge between researchers ( Maryl *et al.*, 2020; Thoden *et al.*, 2017). Frequently, digital tools are applied to speed up the existing (traditional) research methods rather than for methodological innovation ( Gibbs & Owens, 2012, 9).

The need to link the development of infrastructure to thorough user research is visible in recent studies. For instance, the *Survey and Analysis of Basic Social Science and Humanities Research at the Science Academies and Related Research Organisations of Europe* (SASSH) showed the reality of multilingualism across European researchers, thus proving the need for infrastructure that accommodates different languages ( Leathem & Adrian, 2015, 132). There are a number of recent and ongoing projects in which user research is conducted in order to build and enhance research infrastructures such as the Social Sciences & Humanities Open Cloud (SSHOC) ( Barbot *et al.*, 2019, 15–22), and DARIAH ERIC Sustainability Refined (DESIR) ( Tasovac *et al.,* 2018, 9).

In the field of scholarly communication, two current projects that are affiliated with OPERAS are conducting user-research. The Open Scholarly Communication in the European Research Area for Social Sciences and Humanities – Preparation (OPERAS-P), an EU-funded project, has launched a survey on SSH scholarly communication and the challenges of openness, together with focus studies aimed at gaining a more in-depth understanding of communication practices. WP 3, *Co-design and user research*, within the current OPERAS project Transforming Research through Innovative Practices for Linked Interdisciplinary Exploration (TRIPLE) conducted another survey on sources discovery as well as series of interviews with researchers and other stakeholders. They aimed to capture current scholarly discovery practices, and the users’ needs that would be incorporated into building a new platform for finding, accessing, using, and re-using research materials. Indeed, users will continue to be involved and consulted at all phases of the project.

The picture that emerges from these studies shows a rapidly changing communication landscape to which scholars are trying to adapt. Giglia points out that the changing idea of the “scholarly record,” which now also encompasses materials generated in the process, results in the emergence of a more liquid output and blurs the roles of the different actors in scholarly communication ( Giglia, 2019, 142). Currently, sharing practices involve not only concluded research and published outputs but also the *byproducts* and *beta-results* of the research process. This, in turn, results in a wide range of objects that could be communicated, like texts, data, methods, and software ( Bardi, 2019, 4).

The DARIAH survey of scholarly practices and digital needs in the arts and humanities showed that open dissemination channels such as OA publications, repositories, blogs, websites, and scholarly social networks (e.g. Academia.edu and Researchgate) are used frequently by 10–15% respondents and regularly by 35–45% ( Dallas *et al.*, 2017, 4). Yet, even more interesting is the fact that 10% regularly use generic online content services like Slideshare, Flickr, and Youtube for this purpose (ibid.). It is an indicator of the increasing need for new and open avenues of distributing research outputs. Researchers promote their work on social media and engage in less formal discussions about their output using such outlets as Twitter ( Estelle, 2017; Kulczycki, 2013). Online communication tools like WhatsApp are employed to foster communication within the research team ( Estelle, 2017, 7).

The main obstacle to this productive proliferation of new communication practices lies in the fact that these informal communication channels remain invisible to the research assessment system and, consequently, to academic prestige ( Tóth-Czifra, 2019). For instance, research on academic blogging discussed by Brown and Woolston shows that despite general approval for this form of communication, especially among early career-researchers, some doubts remain as to whether it would be considered serious enough by peers, especially when applying for an academic position ( Brown & Woolston, 2018, 137). Therefore, it is crucial for researchers to see new means of communication as being high quality and effective, because otherwise they might not want to use them. The researchers would support a communication model that is “high impact, rigorously refereed, and of good reputation” ( Rowley *et al.*, 2017, 1210). In the next section, we will try to address these issues, sketching out the key areas for the scholarly communication infrastructure in SSH.

## 3. Key areas for the future scholarly communication infrastructure in SSH

### 3.1. Infrastructures for SSH

Shortly before his untimely death, Jon Tennant, a vocal proponent of open science, sketched out its future priorities and challenges (2020), which now constitutes his legacy. What is interesting about his relatively short proposal, published as a blog post, is its scale. Tennant looked not only at the immediate, direct measures for achieving progress, but rather at a complex reconfiguration of scholarly communication institutions that should take place if progress is to be achieved. These priorities entail research evaluation reform, rethinking the role of scholarly publishers, increasing global participation, community building, and creating alternatives to the commercial platforms ( Tennant, 2020). This latter point also consisted of a risk analysis as to “whether or not the scholarly community is truly ready to take on the burden and bureaucracy associated with controlling a global scholarly communication infrastructure” ( Tennant, 2020).

A recent report by the EC Expert Group, dealing with the future of publishing, puts researchers “at the heart of the scholarly communication of the future,” advocating inclusivity in terms of participants, purposes, and methodologies ( Expert Group to the European Commission, 2019, 25). The authors defined the following principles, which articulated their vision of future scholarly communication: maximising accessibility and usability; supporting and expanding the range of contributions; a distributed, open infrastructure; equity, diversity, and inclusivity; community building; promoting high-quality research; facilitating evaluation; promoting flexibility and innovation; and cost-effectiveness ( Expert Group to the European Commission, 2019, 25–29). In the following section we discuss the main areas where such future infrastructure is needed with regard to SSH, and how it can tackle these challenges.

The discussion will be based on the core values of scholarly communication as defined in the Vienna Principles, which were prepared by Open Access Network Austria (OANA). We have taken a similar approach, not treating openness as a goal in itself but rather as a means to achieve broader principles, which are implicitly present in the discussions on open science ( Kraker *et al.*, 2016, 2). Kraker and colleagues have identified the following deficits in the current scholarly communication system: restricted access to scholarly materials and inhibited collaboration opportunities between the various actors due to closed communication; inefficient production, evaluation, and dissemination processes, which do not fully embrace and exploit the possibilities of digital technologies; a lack of transparency in evaluation, and a lack of access to data and contextual material on research that hinders the reproducibility of results; technical and legal restrictions; and a constraining reward structure ( Kraker *et al.*, 2016, 4–5).

Addressing these multiple challenges in a meaningful and effective way requires a coordinated effort by multiple stakeholders; this could be provided by a research infrastructure. As discussed above, the specificity of SSH scholarly communication, its outputs, and practices, calls for dedicated research infrastructures. There are several European Research Infrastructure Consortia attuned to SSH, like DARIAH, focused on arts and humanities (A&H) research data; CLARIN, working with linguistic resources; CESSDA and ESS with social science data; and E-RIHS, ^10^ dedicated to cultural heritage objects. Although scholarly communication remains embedded in the operations of these RIs, none of them address this issue comprehensively. On the other hand, in all disciplines we observe that “[a] growing number of digital publishing initiatives are approaching scholarly communication in new ways and incorporating dynamics of openness, networking, and collaboration into their most basic functions” ( Ren, 2013, 745), which calls for coordinated action to streamline these movements and take advantage of their momentum so that scholarly communication is aligned with researchers needs.

The discussions presented in the sections above have led us to define the following key areas in which an infrastructure can play a vital role in making open scholarly communication a reality in SSH: open access to outputs, discipline-specific genres, evaluation and quality assurance, impact on local societies through multilingualism, scholarly guidance, and the inclusion of various stakeholders. The remainder of this paper addresses the role a research infrastructure could play in these areas in order to assist a systemic change in SSH scholarly communication.

### 3.2. Open access to outputs

The first issue is access. Although its crucial component is mere accessibility, i.e., the free, unrestricted, and sustainable dissemination of knowledge within a community; access should be understood more broadly as allowing for the discoverability and reusability of resources ( Kraker *et al.*, 2016, 7). This strengthens both the efficient and effective identification of resources and scientific dialogue within and between scholarly communities (ibid.).

These principles could easily be illustrated by everyday research practices in SSH. While conducting several systematic literature reviews we repeatedly stumbled over the same telling obstacle. While it is relatively easy to search through the vast collections of commercial databases like Scopus or Web of Science with an elaborated, tailored search string that employs a proximity search for various keywords in selected groups of journals, the situation is dramatically different for the open-access content. Although a very valuable contribution to scholarly content discovery has been delivered by the aggregators for both open publishing (e.g. DOAJ, DOAB) and repository content (e.g. OpenAire, CORE), they provide different search options, rarely allow for full-text search, and do not cover the open content scattered throughout smaller, national or regional infrastructures. In consequence, a comprehensive literature review covering open access papers would have to be conducted through dozens of smaller outlets. As Mounier observes, “[g]iven the multiplicity of dissemination platforms that currently exist, researchers have to browse through too many websites if they want to cover all publications in their field or, alternatively, use Google” ( Mounier, 2018, 304).

Moreover, large commercial databases tend to underrepresent SSH outputs ( Kulczycki *et al.*, 2018); hence, scholars need to resort to web browsing and popular search engines to retrieve interesting content ( Dallas *et al.*, 2017), with all the biases that come with search and personalisation algorithms. The variety of resources encompassing “electronic publications, digital libraries, repositories of full-text papers, algorithms, datasets of scientific data, terminological knowledge bases,” thus requires dedicating “greater efforts to discovering, examining, comparing, and integrating these resources” ( Marcondes, 2012, 73). So, instead of struggling with scholarly content overflow on the web, we need to follow Marcondes suggestion and harness the potential of the digital environment for better content discovery.

This process should run in both directions and also enable researchers to “publish intermediate and relevant products of the research process, i.e. raw data, secondary data, and publications, in a way that they are discoverable, meaningfully interlinked, and re-usable by others” ( Castelli *et al.*, 2013, 155). Modern technology should allow their work to be sustainably stored, discoverable, and easy to reuse. Hence, the scholarly research infrastructure needs to make these scattered resources available for discovery. In addition, gathering and interlinking such materials and metadata strengthens the transparency of research by providing contextual information that helps in the assessment of the source’s credibility ( Kraker *et al.*, 2016, 8).

There is a clear “need for a single discovery platform dedicated to SSH, indexing all types of content (primary sources, publications, grey literature) in different languages and across different countries” ( Mounier, 2018, 304). One step towards this goal was recently made by the Open Access Publishing in European Networks (OAPEN) Library, which moved to a new platform with better browsing options, and improved metadata and API. Another platform is currently being developed in the TRIPLE project, as one of the OPERAS services’ aims is to address these shortcomings by simplifying access to OA materials for researchers and other stakeholders. This platform will be based on the CNRS’s Isidore search engine and will provide the user with a single access point for OA resources, including publications, projects, researchers’ profiles, and data. European diversity is at the heart of the TRIPLE project so it will ensure that SSH research is more discoverable across different cultures and languages: apart from the three languages already managed by ISIDORE (English, French, Spanish), six additional ones will be used: Croatian, German, Greek, Italian, Polish, and Portuguese. Importantly, the initiative is well aligned with the broader EU strategy and will be integrated into EOSC, thus serving as the gateway for SSH open-science outputs in this pan-European endeavour.

### 3.3. Support for SSH genres

A scholarly communication research infrastructure should not restrict, but rather enable, the successful transfer of knowledge in all genres and formats used in a given discipline. This resonates with the European Commission’s ‘holistic approach’ to EOSC, as described earlier, whereby no discipline must be left behind. Hence, we shall embrace disciplinary specificities to successfully attune infrastructures to the actual needs. The results of SSH work are still often mainly published using traditional channels such as monographs and articles ( Bulger *et al.*, 2011), and the infrastructure should cater for these and provide tools for their successful dissemination. There are some differences between how these particular genres are supported by current infrastructures given the stress on journal communication in other disciplines. Monographs are often neglected in terms of OA funding and mandates ( Deville *et al.*, 2019). Also, the SSH publishing landscape should be taken into consideration, as it consists of scattered, smaller publishing houses ( Ferwerda *et al.*, 2016; Tanner, 2016). There are numerous business models and initiatives to support open publication in SSH ( Speicher *et al.*, 2018); hence, the role of infrastructures in this respect encompasses both streamlining the initiatives and also advocating for policy measures that are relevant to diverse outputs.

A good example of the role that infrastructures can play in such endeavours is OpenEdition, which works with a freemium model and allows open access to the full texts of books and articles while delivering premium services, such as other file formats, to libraries, thus providing revenue for smaller publishers who want to disseminate their content through digital means. Another example is Language Science Press, which is supported by a network of cooperating institutions, and provides a platform and technical support for a community of linguist authors, engaging volunteer researchers in the process ( Nordhoff & Kopecky, 2018).

However, an equally important task for research infrastructures is to support scholars in embracing the innovative potential of new technologies. “These potentials include real-time exchange and dissemination, ubiquitous and simultaneous availability of resources, zero marginal cost for dissemination, new workflows, improved reusability of data and results, the ability to process huge volumes of data and new forms of presenting” ( Kraker *et al.*, 2016). This applies to the linking of various outputs together, as already discussed in section 3.2 above, but also to allowing innovative and less formalised genres of communication.

Although digital publishing has been around for more than two decades, we still think of communication in Gutenbergian terms. The publishing process is slow and focused on the final output, and is thus reluctant to accept versioning. Yet, open, digital communication allows for the rapid exchange of outputs, also in formats “that would have been considered unpublishable by traditional publishers” ( Ren, 2013, 74). While an article in a journal *freezes* the research at a certain point, “the Web opens the workshop windows to disseminate scholarship as it happens, erasing the artificial distinction between process and product” ( Priem, 2013, 437). Instead of freezing the outputs, the infrastructure may support the paradigm of the “continual improvement in scholarly publishing” proposed by Juhas *et al.* (2018), whereby a digitally-enabled service would allow for the text to be enriched, and commented on at any point. Instead of thinking about a scientific paper in static terms we can understand it “as a dynamic document evolving in time, which can have different versions and releases, published online, enabling incremental and continual improvement in analogy to software” ( Juhas *et al.*, 2018, 245).

A good example of an innovative genre can be drawn from Open Notebook Science (ONS), which entails providing up-to-date information on research progress by putting the lab notebook online. The audience has access to the raw descriptions of methods, results and research data, and code, which makes the entire research process transparent. *The Open Digital Archaeology Textbook* is an example of such collaborative work; it is integrated with live open code notebooks that can be reused, altered, or extended. A similar genre is living books (for examples, see the series Living Books About Life), an open access publication that is “open to ongoing collaborative processes of writing, editing, updating, remixing, and comment by readers.”

### 3.4. Support for evaluation and quality assurance

Research infrastructures for SSH should support quality assurance and evaluation through transparent peer review practices of the scholarly outputs and metrics used for assessing their impact ( Kraker *et al.*, 2016, 9). Historically speaking, “since the 1960s and 1970s, control of the measures of academic prestige—starting with the management of peer review, and extending to the development of metrics—has been silently transferred from communities of academic scholars to publishing organisations” ( Fyfe *et al.*, 2017, 13). Nowadays, many institutions and countries base performance indicators on those metrics, disregarding biases (e.g. underrepresentation of SSH works, monographs, and critical editions) and differences in citation practices (e.g. in the humanities the impact of research is achieved more slowly and could be measured differently). There is also a risk of bias, as “article-level metrics may also be skewed by the advantages available to big publishers (such as inclusion in key bibliographical databases, more effective marketing and publicity, or the direct ownership of key analytical tools)" (ibid.).

SSH should thus evaluate their own products without delegating—as is common practice in STEM—the selection of metrics and indicators to commercial databases. Research infrastructure can gather data for metrics tailored to SSH, providing guidance, support, and services. The European Commission’s Working Group on Rewards under Open Science argues for using such multi-dimensional criteria in evaluation, as researcher’s “merits, achievements, usefulness are a complex set of different variables, impossible to be summarised by a single figure” ( Working Group on Rewards under Open Science, 2017, 7). The findings of the working group led to the proposing of the Open Science Career Assessment Matrix (OS-CAM), which represents a range of evaluation criteria for assessing open science activities, i.e., publishing datasets according to FAIR principles, adopting quality standards, contributing to public engagement, sharing results through non-academic outlets, and translating research into other languages (ibid. 4–5). Such an evaluation should also incorporate altmetrics in the evaluation process to assess “the wider societal impact of research articles,” which, “in conjunction with citation-based metrics can lead to a clearer picture of societal impact of scientific research” ( Tennant *et al.*, 2016, 7–10). These details could be harnessed by infrastructures, as some repositories already provide altmetrics data for the digital objects they store. Yet, the challenge is to translate them into an instrument for evaluation. Some initiatives, like ImpactStory, are already collecting various data to trace their actual impact ( Priem, 2013, 439). An RI could play a role here by collecting scattered data that is adjusted for evaluation purposes in SSH.

Apart from evaluation and impact measures, RIs can also support the assessment of scholarly quality. Innovative peer review practices, on the other hand, are meant to make the process more transparent, for example, through revealing the names of reviewers in the open-identity review ( Kulczycki *et al.*, 2019a, 3), or to foster the exchange of ideas by making the reviews openly available during the process of the open peer review.

The openness of the review process is ensured by publishing reports alongside articles and by strongly urging, but not necessarily mandating the disclosure of the identity of reviewers.... The review process is turned into a collaborative effort either through the communication among editors and authors, or through initiating discussion within research communities. ( Schmidt & Görögh, 2017, 66)

It could also replace the pre-publication review with the post-publication open peer review, aligned with the abovementioned “continual improvement process,” in the course of which “any researcher can write a peer review of a version of an already published paper or comment the paper, give the paper a rating etc.” ( Juhas *et al.*, 245).

Works published at F1000Research (e.g. Tennant *et al.*, 2016) serve as a good example of such a peer review practice, in which consecutive versions of the paper are reviewed and the comments are published together with the paper. Although this may sound revolutionary, these proposals are actually recreating a scholarly dialogue as is the case of scholarly debates around controversial texts. For instance, *Critical Inquiry* opened a forum discussion around Nan Z. Da’s (2019) article, which stirred the digital humanities community and resulted in many responses that critically assessed the work. Scholars can follow and contribute to a discussion that not only focuses on the given work but also expands its scope, proving that access to actual reviews may be genuinely beneficial for the community. The online version of *Debates in Digital Humanities* ( Gold, 2012) serves a somewhat similar purpose by allowing readers to annotate and discuss the content of articles, thus maintaining the debate not only by presenting different perspectives but also allowing the involvement of readership. Similarly, some book projects, like Kathleen Fitzpatrick’s *Generous Thinking*, or *Exploring Big Historical Data: The Historian’s Macroscope* by Shawn Graham, Ian Milligan and Scott Weingart, use online tools to publish *open drafts* of their work and solicit feedback from the community that could improve the final output.

One of the roles of infrastructure in respect to this lies not only in providing the right procedures and tools to carry out the process, but also in advocating for policy and institutional changes that could recognise and apply the results of these practices. Tennant & Ross-Hellauer (2020) sketched a roadmap for research on peer-review that may identify its shortcomings and biases, leading to the design of shared-data services in this field. Schmidt & Görögh (2017) provided an extensive review of emergent peer review services with such functionalities, for example, Pubpeer.com, a platform for post-publication peer review, where “collaboration between authors, editors and reviewers is strongly encouraged in order to improve the paper and the overall review experience” ( Schmidt & Görögh, 2017, 66). Publons.com, on the other hand, records the peer review contributions of authors and adds it to their Open Researcher and Contributor ID (ORCID) (ibid., 67). There are also tools for open annotation that allow additional commentary layers to be appended on top of content, like PaperHive (repository-based), or hypothes-is (web-based) (ibid., 68-69). The latter tool was recently adapted, as an output of the High Integration of Research Monographs in the European Open Science (HIRMEOS) project (discussed later), so it could annotate digital monographs. However, for such approaches and tools to succeed in transforming our practices, we need to ensure their respectability among the researchers who will use them. Such actions should be integrated with reward practices to make sure that they become a source of prestige in a given discipline.

### 3.5. Impact on local societies through multilingualism

Another potential role for scholarly communication RI is in supporting publications in local languages, which is crucial in SSH as they are often addressed to local communities. The danger of the monopoly of English is also well recognised in open access publishing. For example, according to Shen, who analysed the impact of Chinese publications, only 24% of journals included in the Directory of Open Access Journals (DOAJ) are published in other languages ( Shen, 2017, 2). Academics are under pressure, often amplified by national evaluation policies, not to publish in local languages, which in turn puts non-English-language journals in “danger of losing high-quality academic papers authored by domestic researchers, which will lead to a decline in journal impact and poses a challenge to the survival of such journals” (ibid., 1–2). Such pressures downplay the need for local impact and sustaining knowledge exchange among domestic researchers (ibid., 13). Hence, what is at stake is the future of multilingual scholarly practices and thus the sustainability of local languages as equally valid media for scientific and cultural communication.

Signatories to the Helsinki Initiative on Multilingualism in Scholarly Communication addressed this problem by preparing a set of recommendations for policy-makers, institutions, funders, libraries, and researchers that aimed to “support dissemination of research results for the full benefit of the society,” and “promote language diversity in research assessment, evaluation, and funding systems” ( Federation Of Finnish Learned Societies *et al.*, 2019). These ideas resonate with the postulate of “bibliodiversity,” i.e., “cultural diversity applied to the world of books,” proposed by the International Alliance of Independent Publishers (2018), highlighting “the need to encompass a diversity of languages, scientific areas, publication formats, and actors” ( Leão & Balula, 2019, 1). Jussieu Call for Open Science and Bibliodiversity, an initiative of scientific publishing stakeholders, views this concept as challenging the power relations in scientific communication by “putting an end to the dominance of a small number among us imposing their terms to scientific communities.” In a follow-up call for action, Shearer *et al.* enumerated the barriers to bibliodiversity, such as the already discussed dominance of English, the concentration of infrastructures and services, the limited funding models, and the narrow focus on journal-based policy measures (2020, 5–10).

Bibliodiversity has, therefore, a clear infrastructural dimension: the accessibility, described earlier as the fundament of open-access communication, should embrace access to multilingual content and allow the diversity in the system to be reconstituted (see: Mounier, 2018, 304; Leão & Balulam, 2019, 4; Shearer *et al.*, 2020, 10–11). The Helsinki Initiative explicitly calls for the protection of national infrastructures that publish and disseminate research results of local relevance. Signatories call for the provision of sufficient resources for these initiatives and support for them in maintaining “high standards of quality control and research integrity” ( Federation Of Finnish Learned Societies *et al.*, 2019). A good example of this mission being fulfilled by an infrastructure is provided by SciELO, an electronic library of Brazilian research articles created in response to the underrepresentation of content in Portuguese and Spanish in international databases, and thus in global knowledge exchange. After two decades of operation, it “provides visibility to the journals it hosts in international indexes such as Scopus, WoS, Latindex, and others, resulting in more than 1.5 million COUNTER-certified daily downloads from all over the world in 2017 for the whole platform” ( Mounier, 2018, 301). Redalyc.org, a similar project for Latin America, the Caribbean, Spain, and Portugal, currently hosts over 600 thousand articles from 1.3 thousand journals. Both initiatives signify the need for such infrastructures and exemplify a possible solution.

### 3.6. Scholarly guidance

If scholarly communication is to serve the research community, it has to be led by researchers. The Vienna Principles endorse the idea of the validating progress of scholarly research: “A system of scholarly communication should identify research gaps and highlight fields that need engagement and contribution ... Therefore, [it]should also promote the reproduction and continual validation of existing knowledge” ( Kraker *et al.*, 2016, 10). Thus, communication should be attuned to provide the best possible services that can support the advancement of knowledge. Only a scholarly-led, transparent, and researcher-oriented infrastructure will truly address the existing and emerging needs of scholars of the digital age, by basing its activities on the actual, empirically-evidenced needs of the community, not on the pursuit of commercial revenue.

As Fyfe and colleagues showed, the control over scholarly communication had gradually been handed over to commercial companies and “academics as authors are not yet free to act entirely in the interests of the most efficient system of research communication" (2017, 18). The logic of commercial revenue is often at odds with scientific needs, especially in terms of open access publishing ( Hartley *et al.*, 2018). To be clear, reclaiming scientific communication does not mean excluding commercial players, but rather providing a healthy balance between the commercial interests of publishers, providers, and researchers, which would protect the interests of scholars and smaller players. This would require close cooperation between all stakeholders, such as “governments, funders, universities, learned societies and publishers” ( Fyfe *et al.*, 2017, 19). A good example of such collaboration is the 2.5% Commitment, an initiative that encourages academic libraries to commit this percentage of their budgets to supporting the development of open scholarly content and infrastructures ( Lewis, 2017). Although Neylon (2018) partially agrees with this proposal, he also points out its weaknesses, which sheds more light on the question of cooperation. The first issue is that of coordination mechanisms, which are crucial for the success of the infrastructure and should be community-driven, as “communities that understand and can work with knowledge products are better placed to support them than either the market, or the state” (2018). There needs to be an understanding of the shared cause, as “infrastructures need to be seen as both sustaining and being sustained by the communities that they serve” ( Neylon, 2017b, 8). Second, what emerges from this argument is that fees should be treated as investments, not costs, because they provide “direct benefits to contributors that arise as a side effect of contributing to the collective resource” ( Neylon, 2018). The emerging Invest in Open Infrastructure initiative aims to address these issues by building a recommendation system for funders based on a regular census of infrastructural projects to ensure coordination in the infrastructural response to the scholarly needs of various communities.

As to the benefits for the scholarly community, the HIRMEOS project is an example of an action that could be adopted by a scholarly research infrastructure in the interests of researchers and for the benefit of “small-scale independent partners with limited resources,” allowing them “to cooperate and gain economies of scale by sharing the costs and resource for technical development in order to implement services that are normally accessible only to larger companies who have much greater financial resources and expertise to draw on” ( Mounier, 2018, 304). The project developed a set of services dedicated to identifiers certification, annotation, named-entity recognition, and metrics, that could streamline communication between the resources held by smaller institutions.

### 3.7. Inclusion of various stakeholders

Finally, scholarly communication is efficient only if it encompasses both communication within and beyond the research community. Kraker *et al.* define it as a principle of understandability, entailing adapting the communication for “different stakeholder groups inside and outside of academia, by taking into account specific requirements in order to make it more meaningful and allowing for further involvement and participation" (2016, 8). It is also linked with the principle of collaboration, which “leads to a better understanding of research among stakeholders, and stakeholders can point out research questions that are important to them" (ibid., 8–9). It is even more crucial in the case of SSH, where knowledge legitimation “demands not only scientific peers but also society” ( Kulczycki *et al.*, 2019b, 10).

There are many types of stakeholders at various levels of scholarly research infrastructure, starting from content creators, and progressing to providers and consumers. Such stakeholders as researchers, publishers, libraries, media, non-profit organisations, and companies can participate in all stages of this process. In order to maximise the usefulness, and thus the impact, of the infrastructure it should be inclusive and flexible enough to accommodate the needs of different groups.

 In this respect some lessons could be learned from The Open and Collaborative Science in Development Network (OCSDNet), established in 2015 in order to foster the contribution of open science to achieve developmental goals. The first goal is to build a common language, i.e., enabling a reflective process “around shared principles and goals, to ensure that everyone is striving towards a common objective” ( Hillyer *et al.*, 2017, 29). Second, the authors stress the importance of adjusting goals to suit different stakeholders, as “there is no one-size-fits-all approach to open science, but it is instead a flexible concept that should be adapted to reflect local norms and realities” (ibid., 30). Finally, stakeholders should be empowered by deciding which data should be open to the public (ibid.).

EKT ePublishing, a project developed by the National Documentation Centre in Greece, provides a useful example of how multiple stakeholders can be accommodated by a research infrastructure. The project provided an infrastructure for scholarly eJournals, eBooks, and eProceedings that could be used by non-profit institutional publishers to disseminate their publications ( Nafpliotis *et al.*, 2014). Creating a vast community of stakeholders resulted in increasing researchers’ awareness of modern scholarly communication tools and created a demand from scholarly communities (ibid.,114). Another example is TOME (Toward an Open Monograph Ecosystem), a project that brings together different stakeholders in the United States (i.e., researchers, universities, and libraries) to create a sustainable open monograph ecosystem and open SSH scholarship to a wider readership. TOME recognises the deficiencies in the funding model for monographs and aims to subsidise these outputs through institutionally funded faculty book subsidies. The cooperation, under an ongoing five-year pilot project, is based on the universities providing baseline grants for publications and the publishers committing to producing open-access editions of TOME books. The programme also encourages innovative book formats, enabling the incorporation of multimedia, annotation, and commenting tools.

## Conclusions

We began this article on an optimistic note, recognising the recent change in attitudes towards open access, which could lead to a durable reconfiguration of the scholarly-communication landscape. Let us conclude by addressing some of the threats.

In a recent blog post that addressed open-access policies during the COVID-19 pandemic, Samuel Moore expressed some scepticism as to whether the impact of the current situation will last, as “paywalls have been lifted temporarily, unilaterally and unsystematically, purely in response to a global pandemic crisis. Once this crisis has passed, or at least when publishers deem it to have passed, there is no suggestion that anything other than business as usual will return” ( Moore, 2020). This prediction has already been proven correct, for example through Elsevier’s subsequent announcement that the free access to ‘Coronavirus Research Hub’ will end after 28 October 2020. Moreover, Moore predicts that the crisis may contribute to a further petrification of the communication oligopoly, as smaller publishers may be hit badly by the economic aftermath of the pandemic, which will cripple the budgets in higher education. The situation is becoming even more complicated, because big publishers, as Aspesi and Brand recently observed, may wish to substitute the diminishing subscription revenue with the income from “combined offerings that condition open access to journals upon purchase of other services,” like data analytics or hosting (2020, 574).

This is a wider problem, too. Mariana Mazzucato has recently called for a rethink of public-private partnerships in the wake of the upcoming economic crisis, as “[t]oo often, these arrangements are less symbiotic than [they are] parasitic” ( Mazzucato, 2020). She expressed worries that the global scholarly effort to develop a COVID-19 vaccine may become “yet another one-way relationship in which corporations reap massive profits by selling back to the public a product that was born of taxpayer-funded research” (ibid.). The situation in scholarly communication seems to be awfully similar.

But there is a way out. Moore posits that we should be emancipated “from the idea that knowledge and education can only ever be understood as a commodity and disseminated in a market;” instead, he suggests we need to recognise that “there should be no financial qualification to either accessing or producing such knowledge, and that both could be supported through non-market and economically just means” (2020). And this is precisely where he sees the role of infrastructural projects: to create “commons-based alternatives that point to a better future,” by reinstalling the academic oversight over the scholarly communication (ibid.). As Aspesi and Brand put it, “[t]he time for the academic community to act in coordination is now” (2020, 577).

However, the economy is only one of the complex factors in the scholarly-communication system we have tried to disentangle in this paper. A successful change to, and implementation of, open-science principles in SSH will require a fundamental reconfiguration of the entire landscape that addresses all stakeholders. Kathleen Fitzpatrick provides a tentative list of practices that will have to change: business models; editorial practices; text structures; copyright ownership; archival and preservation practices; relationships between university libraries, presses, technology centers, and academic units; funding models; and the relationship between academia and the surrounding culture ( Fitzpatrick, 2011, 13). And the stakes are high, as she concludes: “As new systems of networked knowledge production become increasingly prevalent and influential online, the university and the scholars who comprise it need to find ways to adapt those systems to our needs, or we will run the risk of becoming increasingly irrelevant to the ways that contemporary culture produces and communicates authority" (ibid.).

As we have argued in this paper, only a scholarly-driven, inclusive research infrastructure for scholarly communication could be up to the task of addressing these aspects, as this comprehensively addresses both structural and systemic frontiers. The numerous papers, projects, and initiatives discussed here prove that scholars have many ideas about how to improve scholarly communication, along with the specific needs that have to be addressed. A dedicated research infrastructure may eventually make this vision a reality.

## Data availability

No data are associated with this article.

## Footnotes

^1 ^E.g. OpenAire Zenodo Community and COVID Gateway, Fairsharing collection, and the European Commission's COVID-19 Data Portal.

^2 ^E.g. OpenBookPublishers.

^3 ^Many thanks to Marina Angelaki (EKT), Pierre Mounier (OpenEdition), Valérie Schafer (C ^2^DH), and Lars Wieneke (C ^2^DH) for their help in grasping the specificities of OA solutions and the scholarly communication landscape in different European countries.

^4 ^See more: http://www.snf.ch/en/researchinFocus/newsroom/Pages/news-200409-national-strategy-on-open-data.aspx.

^5 ^See: https://ec.europa.eu/research/openscience/index.cfm?pg=open-science-cloud

^6 ^For a general introduction to DH see: ( Gold, 2012; Schreibman *et al.*, 2016)

^7 ^For discussion see: ( Borek *et al.*, 2016).

^8 ^For discussion see: ( Pertsas & Constantopoulos, 2017)

^9 ^For further elaboration of this duplicity using the example of contemporary historians, see: ( Maryl *et al.*, 2019, chap. 5)

^10^ Digital Research Infrastructure for the Arts and Humanities (DARIAH); European Research Infrastructure for Language Resources and Technology (CLARIN); Consortium of European Social Science Data Archives (CESSDA); European Social Survey (ESS); European Research Infrastructure for Heritage Science (E-RIHS).
