# Supplementary material for: The case for an inclusive scholarly communication infrastructure for social sciences and humanities
Source: F1000Res. 2020 Oct 22;9:1265. [Version 1] doi: 10.12688/f1000research.26545.1 (PMC7816277; doi:10.12688/f1000research.26545.1)
Supplement: Peer review history

# Peer review: Reviewer response for version 1

**DOI:** 10.5256/f1000research.29305.r77402

**Published:** 2021-01-19

**Authors:** Amy Brand

**Affiliations:** 1 The MIT Press, Cambridge, MA, USA

I applaud Maryl, Błaszczyńska, Szulińska, and Rams for their extremely thorough literature review on scholarly communication infrastructure across the sciences, social sciences and humanities. I learned a tremendous amount from reading the article and approve it for indexing in F1000Research. That said, I believe the article could be made more concise and more forceful than it is currently. As it stands, the article is a description of what’s broken and a statement of need for change, stopping short of providing actionable proposals or a theory of how transformation will proceed. 

 To the extent that this article does sketch a vision of the target state of scholarly communications infrastructure, that vision is one based mostly on coordinated planning and centralized management of some sort, even while scholar-led. I remain skeptical about the likelihood of success of monolithic solutions, given what we know about the important role that entrepreneurship plays in innovation, and about how universities and other parts of the research ecosystem function. (I am aware that US research universities are more operationally decentralized than European universities, and that definitely colors my own views.) 

 Most of this long, ambitious, and at times meandering article is spent reviewing the current state of scholarly communications infrastructure on an international scale, and making the case for an alternative model, one that supports the values of diversity, inclusivity, and multilingualism, and is researcher governed as opposed to market driven. All to the good thus far. However, the reader is then left wanting suggestions for actionable next steps. What form will real progress take, and where will needed innovations come from? Do the values underpinning open knowledge and tools necessarily run counter to competition-based innovation and distributed models of change?

 If the F1000’s editors and article authors are inclined to go further, I propose that the authors summarize the literature review here more concisely, and devote more space to spelling out how to make progress, however gradual, towards a researcher-driven, inclusive infrastructure for open science in the social sciences and humanities (SSH). From the authors’ perspectives, this may be another article entirely — and that’s fine —  but I’ll lay out my own thoughts and questions here in order to help guide potential revisions or future work.

 First, I take issue with the implicit premise here that, because the natural sciences have advanced further and faster than SSH fields towards open access and enriched knowledge infrastructures, models in the sciences are the right ones to follow for SSH. If models of effective scholarly communication in SSH are inherently more diverse and inclusive, as the authors substantiate in their review, then it may be that SSH communities can lead the “harder” sciences in defining at least some new models and infrastructures. For example, why would gold open access be an aspiration for SSH when we’ve seen the problems it has perpetuated in the sciences? And perhaps the more qualitative ways in which we evaluate excellence and prestige in SSH knowledge production have advantages over quantitative citation-based methods that natural science communities can learn from.

 Next, the paper could benefit from a clear definition of scholarly communications infrastructure early on. The authors devote lots of space in this paper to defining scholarly communications within the context of formal and informal communication systems broadly defined, but wait until section 1.3 (page 8) to supply the European Commission’s definition of research infrastructures. So too, that definition stops short of a full depiction of well established scholarly communications infrastructures. In addition to physical and computational edifices and publishing platforms writ large, these include persistent identifier systems such as Crossref and ORCID, metadata frameworks and other technical standards to enable interoperability among systems, content sharing norms such as Creative Commons licenses, and even business models themselves.

 When you embrace a broader definition of knowledge infrastructures, it expands your levers of change. If you describe the challenge in an entirely top-down way — that, for example, in order to effect desired transformation in scholarly communication you must convince the powers that be to tear down old, expensive edifices and cooperate in designing and funding the construction of new, expensive edifices — you may be setting yourself up for many years of frustration.

 Yes, fixing scholarly communications is a large and complex challenge, with multiple stakeholders, and change will take time. But I would urge the authors to investigate the question of to what extent we can let’s accelerate the process by directly giving researchers, societies and mission driven publishers tools to help drive it from the ground up, as it were. What do I mean by that? Standards like the NISO CRediT taxonomy ( http://credit.niso.org/) for representing contributor roles in multi-authored works enable scholars to describe their contributions to scholarship in enriched ways. If we empower scholars with new impact narratives, aren’t we enabling change in academic evaluation that wouldn’t be possible otherwise? I think we are, and believe that seemingly small advances like growing the use of alternative metrics, expanding which scholarly outputs are assigned DOIs, what contributions and works are tracked by ORCID, etc. amount to a powerful enabler of desired change. It’s all infrastructure. Similarly, creating publisher-independent signals of quality and rigor, as proposed in our Peer Review Transparency work https://www.prtstandards.org/report, helps lay the foundation for academic evaluation processes that are less dependent on publisher brands and traditional publication genres to signal contribution and prestige.

 Another grassroots way to increase the variety of “signals” that inform our assessment of scholarly contribution and excellence is via launching new open access publications. There are several examples of SSH outputs and genres in the article. But I am thinking here of new publications like *Reviews in Digital Humanities*, “a pilot of a peer-reviewed journal that facilitates scholarly evaluation of digital humanities work and its outputs. This may include, but is not limited to: digital archives, multimedia or multimodal scholarship, digital exhibits, visualizations, digital games, digital tools, and digital projects… The journal responds to the challenge of the growth of the number and scale of digital projects outpacing review opportunities in existing journals. As such, it intervenes by bridging a gap in the evaluation of digital projects by arranging for peer review of digital scholarship.” (See https://reviewsindh.pubpub.org/about).

 Another very promising “bottom up” approach to change is through the systems that universities use to track and report on faculty activities. Expanding the catalog of works in such systems to include pre-prints, non-traditional publication outputs and genres, or informal communications such as blogs is an indirect but powerful way of deconstructing the institutional norms that hold back change in scholarly communications. Such faculty activity reporting systems can also be directly integrated with institutional repositories (Hanrath, 2016 ^1^). 

 Infrastructure is a big, weighty concept and tends to be talked about in scholarly communication circles, including in this excellent paper, in terms of top-down change and centralized organization and funding. I believe distributed innovation and distributed institutional investment are key to the sustainability of new scholar-led infrastructures. I’ve tried above to spark awareness of some complementary bottom-up approaches to these challenges, colored by admittedly deeper knowledge of US than European academia. I hope these reflections prove helpful to the authors and the readers of this article.

Is the topic of the opinion article discussed accurately in the context of the current literature?

Yes

Are arguments sufficiently supported by evidence from the published literature?

Yes

Are all factual statements correct and adequately supported by citations?

Yes

Are the conclusions drawn balanced and justified on the basis of the presented arguments?

Yes

Reviewer Expertise:

Scholarly communications; academic publishing

I confirm that I have read this submission and believe that I have an appropriate level of expertise to confirm that it is of an acceptable scientific standard.

## References

[1] : Integrating a Faculty Activity Reporting System with an Institutional Repository in Support of Open Access. *Open Repositories 2016, Dublin, Ireland*.2016;

# Peer review: Reviewer response for version 1

**DOI:** 10.5256/f1000research.29305.r73616

**Published:** 2020-11-09

**Authors:** Ioana Galleron

**Affiliations:** 1 UFR Littérature, linguistique, didactique, Université Sorbonne Nouvelle, Paris, France

Starting from an observation of the recent changes in the scholarly communication system (multiplication of forms of engagement due to the multiplication of on-line communication tools and to the digitisation of resources), this paper advocates in a convincing way for the development of an integrated research infrastructure dedicated to the SSH. Such a platform would facilitate the discovery and the reuse of scientific publications and data, would contribute to preserving linguistic, methodological and epistemic diversity, especially in the humanities, and would stimulate the creation and/ or the expansion of interdisciplinary communities. The needs of SSH scholars with regards to scholarly communication are attentively scrutinized and well understood, both through personal engagement of the authors in user-research within the OPERAS project, and thanks to the perusal of an extensive bibliography.

 However, the paper seems to me, for the moment, insufficiently focused. The first part (p. 3 to 11) appears to propose a rather theoretical discussion about the gaps impeding a streamlined, multi-lingual, multi-purposed scholarly communication, but the second part (p. 12 and subsequent), abandons the “looking at the future” perspective and sounds much more as a kind of monitoring report on the advances towards an integrated platform for scholarly communication. This second part is, indeed, much more focussed on existing projects, products or initiatives (the TRIPLE project, the OpenEdition endeavour p. 12, the HIRMEOS annotation tool p. 13 and 15, EKT ePublishing p. 15, etc.), the larger part of them being under development within OPERAS realm. This oscillation between “should” and “has been done” is somewhat confusing for the reader, and could be avoided through putting forward the second focus from the very beginning, as an opinion paper allows to do.

 I also recommend to shorten the background part of the paper, that can be deemed in places as breaking open doors (such as the role of communication as enabler of science, or the negative effects of scholarly publishing industry), to the detriment of elaborating more on the solutions provided by OPERAS and some other providers. As an example, the difference between the TRIPLE project and the OAPEN Library should be explored in more depth, so as to avoid giving the impression of competing products that tackle the same issue, or to justify the need for such a competition. Also, when the authors discuss the post-publication peer-review and the commentary layers appended on already published content (p. 13-14), it is unclear what the contribution of the proposed platform would be. Solutions seem to be already in place, where is the problem and what’s the innovative idea we are talking about here? More generally, the discussion about the challenges related to the streamlining of fragmented initiatives for new forms of scholarly communication could be more developed.

 It may also be useful to revise the discussion about the proposed platform contribution to research evaluation. Too many perspectives and ideas are conflated here, as shown by the following sentence that artificially separates evaluation (probably reduced to “reading of metrics”) and quality: “apart from evaluation and impact measures, RI can also support the assessment of scholarly quality”. The proposed (or existing but incomplete) platform is presented as answering a need (give visibility to alternative communication forms), but also as a lobbying tool that may ultimately result in imposing unwanted obligations to the scholarly community (“blog, tweet or perish”?). In advocating for taking into account all the forms of scholarly communication in career evaluation, the paper underestimates the difficulty for the scholarly communities to think about acceptable standards and rewards for such activities if they are not to become just a form of “posh” or “negatively oriented modern research”. ^1^

 Finally, some compelling affirmations are embraced without further discussion. Undoubtedly, the idea of a publication as a “dynamic document evolving in time” is stimulating. Still, it may be worthwhile to look at what happened in the field of scholarly editing, where the digital format and the TEI freed the scholars 30 years ago from the “frozen document” paradigm. As Elena Pierazzo puts it, this led to the creation of a culture of “perpetual prototype(s)”. Nowadays, platforms and consortia struggle with a form of publishing procrastination, with many resources existing in a digital format, but unavailable for the large public, because deemed insufficiently “scholarly” for publication. Alternatively, the “fluid” model may stimulate “premature” publications, whose improvement is promised but never realised for lack of time or other (good) reasons. Therefore, “formal” publication model still has some clear advantages, and I invite the authors of the paper to reflect more on the place it may take in their comprehensive communication eco-system, and on the links, the complementary or the tensions it may have with the other forms of communication.

Is the topic of the opinion article discussed accurately in the context of the current literature?

Yes

Are arguments sufficiently supported by evidence from the published literature?

Yes

Are all factual statements correct and adequately supported by citations?

Yes

Are the conclusions drawn balanced and justified on the basis of the presented arguments?

Yes

Reviewer Expertise:

Digital text analysis, French Literature, Research Evaluation in the SSH

I confirm that I have read this submission and believe that I have an appropriate level of expertise to confirm that it is of an acceptable scientific standard.

## References

[1] : Humanities Scholars’ Conceptions of Research Quality.2016; 10.1007/978-3-319-29016-4_5 43-69 10.1007/978-3-319-29016-4_5
